# Maintenance of low driving pressure in patients with early acute respiratory distress syndrome significantly affects outcomes

**DOI:** 10.1186/s12931-021-01912-8

**Published:** 2021-12-15

**Authors:** Hui-Chun Chang, Chung-Han Ho, Shu-Chen Kung, Wan-Lin Chen, Ching-Min Wang, Kuo-Chen Cheng, Wei-Lun Liu, Han-Shui Hsu

**Affiliations:** 1 Institute of Emergency and Critical Care Medicine, School of Medicine, National Yang Ming Chiao Tung University, No. 155, Sec. 2, Linong St. Beitou Dist., Taipei, 11221 Taiwan; 2 Department of Respiratory Therapy, Chi Mei Medical Center, Liouying, Tainan, Taiwan; 3 Department of Medical Research, Chi Mei Medical Center, Tainan, Taiwan; 4 Department of Information Management, Southern Taiwan University of Science and Technology, Tainan, Taiwan; 5 Department of Internal Medicine, Chi-Mei Medical Center, Liouying, Tainan, Taiwan; 6 Department of Internal Medicine, Chi-Mei Medical Center, Tainan, Taiwan; 7 School of Medicine, College of Medicine, Fu Jen Catholic University, No.510, Zhongzheng Rd., Xinzhuang Dist., New Taipei City, 242062 Taiwan; 8 Division of Critical Care Medicine, Department of Emergency and Critical Care Medicine, Fu Jen Catholic University Hospital, Fu Jen Catholic University, New Taipei City, Taiwan; 9 Division of Thoracic Surgery, Department of Surgery, Taipei Veterans General Hospital, Taipei, Taiwan

**Keywords:** Acute respiratory distress syndrome, Driving pressure, Lung-protective ventilation strategy, Outcome

## Abstract

**Background:**

Driving pressure (∆P) is an important factor that predicts mortality in acute respiratory distress syndrome (ARDS). We test the hypothesis that serial changes in daily ΔP rather than Day 1 ΔP would better predict outcomes of patients with ARDS.

**Methods:**

This retrospective cohort study enrolled patients admitted to five intensive care units (ICUs) at a medical center in Taiwan between March 2009 and January 2018 who met the criteria for ARDS and received the lung-protective ventilation strategy. ∆P was recorded daily for 3 consecutive days after the diagnosis of ARDS, and its correlation with 60-day survival was analyzed.

**Results:**

A total of 224 patients were enrolled in the final analysis. The overall ICU and 60-day survival rates were 52.7% and 47.3%, respectively. ∆P on Days 1, 2, and 3 was significantly lower in the survival group than in the nonsurvival group (13.8 ± 3.4 vs. 14.8 ± 3.7, *p* = 0.0322, 14 ± 3.2 vs. 15 ± 3.5, *p* = 0.0194, 13.6 ± 3.2 vs. 15.1 ± 3.4, *p* = 0.0014, respectively). The patients were divided into four groups according to the daily changes in ∆P, namely, the low ∆P group (Day 1 ∆P < 14 cmH_2_O and Day 3 ∆P < 14 cmH_2_O), decrement group (Day 1 ∆P ≥ 14 cmH_2_O and Day 3 ∆P < 14 cmH_2_O), high ∆P group (Day 1 ∆P ≥ 14 cmH_2_O and Day 3 ∆P ≥ 14 cmH_2_O), and increment group (Day 1 ∆P < 14 cmH_2_O and Day *3* ∆P ≥ 14 cmH_2_O). The 60-day survival significantly differed among the four groups (log-rank test, *p* = 0.0271). Compared with the low ΔP group, patients in the decrement group did not have lower 60-day survival (adjusted hazard ratio 0.72; 95% confidence interval [CI] 0.31–1.68; p = 0.4448), while patients in the increment group had significantly lower 60-day survival (adjusted hazard ratio 1.96; 95% CI 1.11–3.44; p = 0.0198).

**Conclusions:**

Daily ∆P remains an important predicting factor for survival in patients with ARDS. Serial changes in daily ΔP might be more informative than a single Day 1 ΔP value in predicting survival of patients with ARDS.

## Background

Acute respiratory distress syndrome (ARDS) is a severe disease with a high mortality rate (range 35–45%) [1]. The lung tissue of patients with ARDS shows diffuse pathological changes [2], and alveolar destruction causes gas exchange disorders that induce hypoxemia [3]; most patients usually receive intubation owing to hypoxemic respiratory failure, and they require mechanical ventilation [4].

Several therapeutic strategies that may assist in the treatment of ARDS have been proposed, such as the lung-protective ventilation strategy, lung recruitment maneuvers, prone positions, and extracorporeal membrane oxygenation (ECMO) [5]. Several large randomized clinical studies have confirmed that the lung-protective strategy is still the mainstream treatment for ARDS [4–7]; however, no lung physiological parameter can predict mortality. Driving pressure (∆P), proposed in 2015 [8], is a simple calculation formula that can reflect the true pressure condition of the lung due to pathological changes and ventilator settings. Several experiments have confirmed that lung stress and transpulmonary pressure have a positive correlation with ∆P [9, 10]. Other studies have pointed out that the use of dynamic ∆P to predict mortality in patients with ARDS using ECMO yields similar results [11].

However, extensive clinical experience has shown that patients with ARDS often have hemodynamic instability on the 1st day of diagnosis. These patients are usually administered fluid challenge, vasopressors, sedatives, or even muscle relaxants to maintain their hemodynamics [12, 13], all of which affect oxygenation and ventilator settings. In previous studies, ∆P was only assessed on the 1st day of diagnosis of ARDS to predict mortality [8, 11, 14, 15]. No study has reported on the correlation between serial changes in ∆P and the survival of patients with ARDS. Therefore, we assumed that rather than just monitoring Day 1 ∆P, the assessment of serial changes in daily ∆P would better predict the outcomes of patients with ARDS.

## Methods

This single-center, retrospective cohort study was entirely conducted in five intensive care units (ICUs) of Chi Mei Medical Center, Liouying, Taiwan, with a total of 62 adult ICU beds. The study was approved by the Institutional Review Board for Human Research (Chi-Mei IRB No. 10604-L04), and the need to obtain informed consent was waived owing to the retrospective nature of the study.

### Study population

We analyzed patients with ARDS who received intubation and the lung-protective ventilation strategy between March 2009 and January 2018. ARDS was defined and categorized based on the Berlin definition: mild (arterial partial pressure of oxygen to fraction of inspired oxygen (PaO_2_/FiO_2_ ratio) 201–300 mmHg), moderate (PaO_2_/FiO_2_ ratio 101–200 mmHg), and severe (PaO_2_/FiO_2_ ratio ≤ 100 mmHg). Day 1, Day 2 and Day 3 was defined as the 1st day, 2nd day and 3rd day after diagnosed ARDS, that the above criteria were satisfied [1]. Patients aged < 20 years, patients who were pregnant, patients who had received the lung-protective ventilation strategy within 3 days of the diagnosis of ARDS, patients with incomplete mechanical ventilation parameters, and patients with missing arterial blood gas data for more than two occasions were excluded from the study. All patients were sedated and ventilated with the volume-controlled mode and a tidal volume (Vt) setting of 6–8 mL per kg of ideal body weight (IBW) throughout the study protocol.

### Physiological measurements and outcomes

From patient charts, patient data were collected and analyzed, including age, sex, IBW, underlying disease, and Acute Physiology and Chronic Health Evaluation II score. The first arterial blood gas was recorded after the diagnosis of ARDS and before the use of the lung-protective strategy on Days 1, 2, and 3. Ventilator settings included Vt, respiratory rate, positive end-expiratory pressure (PEEP), FiO_2_, plateau pressure (Pplateau), lung compliance, and ∆P (Pplateau − PEEP). Pplateau was calculated as an inspiratory pause of 0.5–1 s by a respiratory therapist. All ventilator parameters were recorded before obtaining daily arterial blood gas, and data with large fluctuations, such as after suction, bronchoscopy, or any transfer, were avoided. Information on the dates of ICU admission, diagnosis, and death was also recorded to calculate the length of hospital and ICU stays. Information regarding the use of other rescue treatments, such as lung recruitment maneuver, prone positions, and ECMO, was also recorded.

The primary outcome was the association of ventilation parameters collected during the first 3 days after an ARDS diagnosis with assisted ventilation and 60-day survival.

### Statistical analyses

Continuous variables are presented as the mean ± standard deviation, and categorical variables are presented as frequencies (percentages). Student’s *t* test or the Mann–Whitney *U* test was used to compare the differences in the distributions of continuous variables between survivors and nonsurvivors. Pearson’s chi-square test or Fisher’s exact test was used to compare the differences in the distributions of categorical variables between survivors and nonsurvivors. The possible risk factors for ICU mortality at 60 days were estimated using a Cox proportional hazards regression model. Kaplan–Meier analysis was used to draw cumulative survival curves, and the log-rank test was used to compare the differences. A p value of < 0.05 was considered statistically significant, and all analyses were performed using the statistical software SPSS, version 20.0 (IBM Corp., Armonk, NY, USA).

## Results

**Fig. 1:**
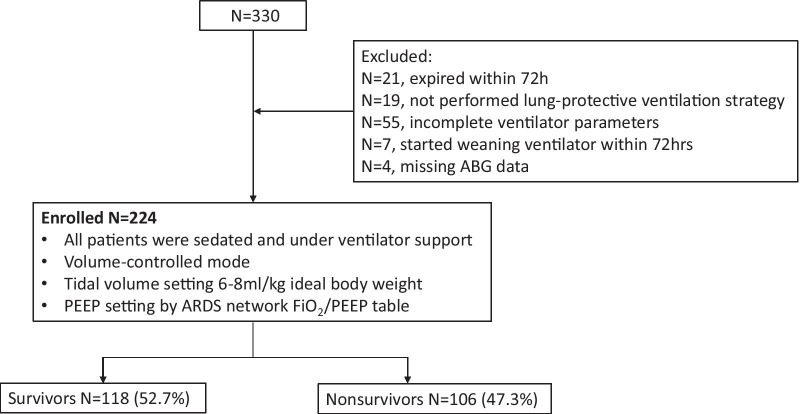
Flowchart of the study. *ABG* arterial blood gas; *ARDS* acute respiratory distress syndrome; *PEEP* positive end-expiratory pressure; *FiO*_*2*_ fraction of inspired oxygen

A total of 330 patients diagnosed with ARDS were admitted to our ICUs and received mechanical ventilation during the study period. Figure 1 shows the study flowchart. A total of 224 patients were finally analyzed, and the overall ICU survival rate was 52.7%. The clinical characteristics and demographic data of the patients are presented in Table 1. Among the 224 enrolled patients, 209 (93.3%) were classified as having moderate to severe ARDS, 90 (40.2%) received a lung recruitment maneuver, 6 (2.7%) were treated in the prone position, and 8 (3.5%) received ECMO support. Between the 106 nonsurvivors and 118 survivors, the nonsurvivors had a significantly lower ARDS Day 3 PaO_2_/FiO_2_ ratio (173.6 vs. 200.4; p = 0.009) and a shorter length of hospital stay (16.7 vs. 37 days; p < 0.001) than the survivors; however, no significant difference in age, sex, disease severity, comorbidities, ARDS etiology, ARDS category, treatment strategy, or Day 1 and Day 2 PaO_2_/FiO_2_ ratios was observed between the two groups.

**Table 1:** Baseline characteristics of patient with ARDS

Parameter	All patients N = 224	Survival N = 118	Non-survival = 106	p value
Age, mean ± SD	67	65.4 ± 17	68.8 ± 14.7	0.114
Male	160	83	77	0.703
ARDS etiologies, N (%)				
Pneumonia	168 (75)	90 (76.3)	78 (73.6)	0.643
Sepsis	21 (9.3)	11 (9.3)	10 (9.4)	0.422
Other causes	35 (15.6)	17 (14.4)	18 (17)	0.596
Disease severity				
APACHE II score	23.2 ± 7.1	22 ± 6.8	24.5 ± 7.3	0.09
Comorbidities, N (%)				
Diabetes mellitus	90 (40.2)	51 (43.2)	39 (36.8)	0.327
Chronic heart disease	24 (10.7)	11 (9.3)	13 (12.3)	0.477
Chronic pulmonary disease	34 (15.2)	17 (14.4)	17 (16)	0.734
Chronic liver disease	36 (16.1)	15 (12.7)	21 (19.8)	0.149
Chronic renal disease	28 (12.5)	13 (11)	15 (14.2)	0.149
Malignancy	51 (22.8)	23 (19.5)	28 (26.4)	0.149
ARDS category, N^†^ (%)				
Mild	14 (6.3)	8 (6.8)	6 (5.7)	0.776
Moderate	150 (67)	81 (68.6)	69 (65)	
Severe	59 (26.3)	29 (24.6)	30 (28.3)	
Rescue treatment, N (%)				
Recruitment maneuver	90 (40.2)	51 (43.2)	39 (36.8)	0.327
Prone position	6 (2.7)	1 (0.8)	5 (4.7)	0.073
ECMO	8 (3.5)	3 (2.5)	5 (4.7)	0.381
Duration, mean ± SD				
Length of ICU	14.1 ± 7.5	14.9 ± 6.9	13.1 ± 8	0.069
Length of in-hospital	27.4 ± 22.3	37 ± 24.5	16.7 ± 12.6	< 0.001
Arterial blood gas, mean ± SD				
Day 1				
PaO_2_/FiO_2_ ratio^†^	151.7 ± 61.7	158.2 ± 68.7	144.4 ± 51.9	0.096
PaCO_2_^†^	39.5 ± 10.6	38.7 ± 9.8	40.3 ± 11.5	0.27
Day 2				
PaO_2_/FiO_2_ ratio	173.3 ± 67.3	180.8 ± 73.6	164.8 ± 58.6	0.075
PaCO_2_	40.1 ± 33.2	41.9 ± 45.1	38.2 ± 7.4	0.403
Day 3				
PaO_2_/FiO_2_ ratio	187.9 ± 76.7	200.4 ± 78.6	173.6 ± 72.2	0.009
PaCO_2_	37.5 ± 7.7	36.8 ± 7.7	38.2 ± 7.6	0.189
Ventilator parameters, mean ± SD				
Day 1				
Vt (mL/kg IBW)	7.4 ± 0.89	7.5 ± 0.8	7.3 ± 0.9	0.0701
PEEP (cmH_2_O)	12.8 ± 2.6	12.5 ± 2.4	13.1 ± 2.8	0.0864
Day 2				
Vt (mL/kg IBW)	7.3 ± 1.1	7.4 ± 0.9	7.1 ± 1.2	0.0965
PEEP (cmH_2_O)	12.4 ± 2.5	12.1 ± 2.4	12.7 ± 2.5	0.0929
Day 3				
Vt (mL/kg IBW)	7.3 ± 1	7.4 ± 0.8	7.2 ± 1.1	0.1421
PEEP (cmH_2_O)	12 ± 3	11.4 ± 2.7	12.6 ± 3.2	0.0019

Parameter data are presented as mean ± standard deviation

*ARDS* acute respiratory distress syndrome; *APACHE* acute physiologic and chronic health evaluation; *ECMO* extra-corporeal membrane oxygenation; *PaO*_*2*_ arterial partial pressure of oxygen; *FiO*_*2*_ fraction of inspired oxygen; *PaCO*_*2*_ arterial partial pressure of carbon dioxide; *IBW* ideal body weight; *Vt* tidal volume; *PEEP* positive end-expiratory pressure

^†^Data available in 223 patients

**Fig. 2:**
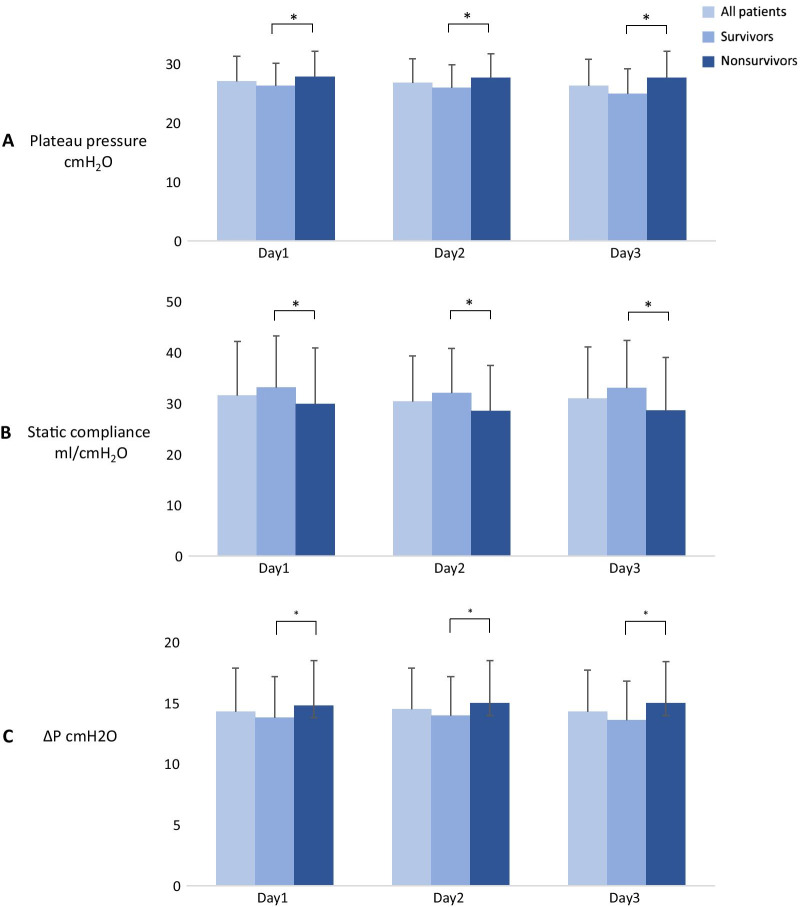
Plateau pressure (**A**), static compliance (**B**), and driving pressure (ΔP) (**C**) in all patients, survivors, and nonsurvivors on Day1, Day2, Day3. *p < 0.05

We compared the 60-day survival between survivors and nonsurvivors to evaluate the associations between static compliance, Pplateau, and ∆P. On Day 1, survivors and nonsurvivors significantly differed in static compliance (33.2 ± 10.1 vs. 29.9 ± 11, *p* = 0.0204), Pplateau (26.3 ± 3.9 vs. 27.9 ± 4.3, *p* = 0.0033), and ∆P (13.8 ± 3.4 vs. 14.8 ± 3.7, *p* = 0.0322). On Day 2 & Day 3, survivors had higher static compliance (32.1 ± 8.7 vs. 28.6 ± 9, *p* = 0.003; 33.1 ± 9.4 vs. 28.6 ± 10.5, *p* = 0.0008, respectively), lower Pplateaus (26.0 ± 3.9 vs. 27.7 ± 4.1, *p* = 0.0032; 25.0 ± 4.2 vs. 27.7 ± 4.5, p < 0.001, respectively), and lower ∆Ps (14.0 ± 3.2 vs. 15.0 ± 3.5, *p* = 0.0194; 13.6 ± 3.2 vs. 15.1 ± 3.4, *p* = 0.0014, respectively) than nonsurvivors (Fig. 2).

**Fig. 3:**
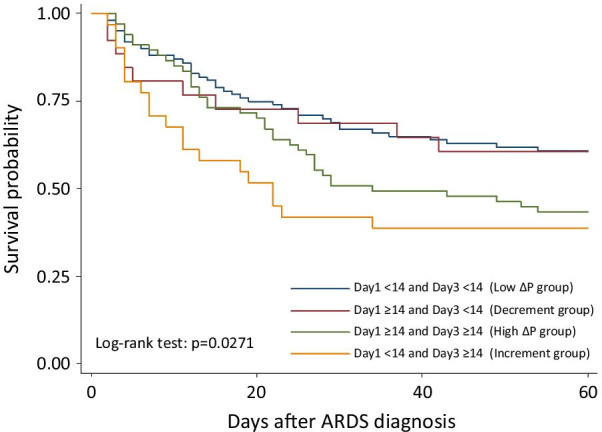
Kaplan–Meier probability of survival from the day of ARDS diagnosis to Day 60. Patients are stratified in for subgroups according to the levels of driving pressure (∆P) on Day 1 & Day 3

To evaluate the serial changes in ∆P during early ARDS, patients were divided into four groups according to serial changes in ∆P, namely, the low ∆P group (Day 1 ∆P < 14 cmH_2_O and Day 3 ∆P < 14 cmH_2_O), decrement group (Day 1 ∆P ≥ 14 cmH_2_O and Day 3 ∆P < 14 cmH_2_O), high ∆P group (Day 1 ∆P ≥ 14 cmH_2_O and Day 3 ∆P ≥ 14 cmH_2_O), and increment group (Day 1 ∆P < 14 cmH_2_O and Day 3 ∆P ≥ 14 cmH_2_O). The 60-day survival significantly differed among the four groups (log-rank test, p = 0.0271) (Fig. 3). Compared with the low ∆P group, no significant differences in 60-day survival were observed in the decrement group (adjusted hazard ratio [aHR] 0.72; 95% confidence interval [CI] 0.31–1.68; *p* = 0.4448) and the high ∆P group (aHR 1.02; 95% CI 0.51–2.05; *p* = 0.9475). However, patients in the increment group had significantly lower 60-day survival (aHR 1.96; 95% CI 1.11–3.44; *p* = 0.0198) (Table 2).

**Table 2:** Cox regression analysis of driving pressure (∆P) associated with 60-day mortality in ARDS patients

Patient groups	Crude HR (95% CI)	p value	Adjusted* HR (95% CI)	p value
Day1 < 14 and Day3 < 14 (low ∆P)	1.00 (ref.)		1.00 (ref.)	
Day1 ≥ 14 and Day3 < 14 (Decrement)	1.07 (0.53–2.13)	0.8584	0.72 (0.31–1.68)	0.4448
[Day1 ≥ 14 and Day3 ≥ 14 (High ∆P)	1.59 (1.02–2.48)	0.0425	1.02 (0.51–2.05)	0.9475
Day1 < 14 and Day3 ≥ 14 (Increment)	2.10 (1.21–3.64)	0.0081	1.96 (1.11–3.44)	0.0198

*∆P* driving pressure; *ARDS* acute respiratory distress syndrome; *HR* hazard ratio; *CI* confidence interval

*Adjusted by age, gender, Day 1 driving pressure, Day 1 compliance and Acute Physiology and Chronic Health Evaluation (APACHE) II score

## Discussion

To the best of our knowledge, this is the first observational study to reveal the effects of maintaining low ∆P in early ARDS and its significant association with improved 60-day survival. The main findings of the present study were as follows: (1) in the increment group, for patients whose ∆P could not be maintained at < 14 cmH_2_O in the early phase of ARDS, the mortality rate was significantly higher than that in the low ∆P group and (2) If ∆P could be controlled below 14 cmH_2_O in patients with early ARDS, even though the ∆P on Day 1 was high, the 60-day mortality was similar to that observed in the low ∆P group.

In 1998, Amato et al. reported on the lung-protective ventilation strategy for ARDS, and thereafter, many studies have proven that this strategy can improve the survival of patients with ARDS. In the same study, ∆P was first reported in patients with ARDS [7]. In a secondary analysis study of multiple independent variables, decreases in ∆P were strongly associated with the increased survival of patients with ARDS [8]. Several studies that have focused on the use of ∆P to predict the mortality of patients with ARDS have revealed similar results [4, 11, 15, 16]. ∆P during mechanical ventilation is significantly related to stress forces in the lung. Chiumello et al. suggested that lung stress is associated with ∆P, which, based on the different levels of PEEP, can help detect overstress of the lung. This study found that the optimal cut-off point for ∆P was 15 cmH_2_O when the lung stress reached 24–26 cmH_2_O. The study also confirmed that tidal volume has a very low correlation with lung stress [9]. Baedorf Kassis et al. used an esophageal balloon to confirm the close correlation between the respiratory system and transpulmonary ∆P [10]. High ∆P during controlled or pressure support ventilation is associated with worse long-term outcomes regarding pulmonary function and structure, even in patients who receive the lung-protective ventilation strategy [17, 18]. Baedorf Kassis et al. suggested that ventilation strategies that lead to decreased respiration and transpulmonary ∆P at 24 h could be associated with improved patient survival [10]. Therefore, ∆P might be a useful treatment target during mechanical ventilation in patients with early ARDS [17, 18]. Pereira Romano et al. performed a pilot study that aimed to achieve a ∆P ≤ 10 cmH_2_O in patients with early ARDS and proved that a driving pressure-limited strategy is feasible to achieve this goal [19]. Therefore, in patients with ARDS, a ventilator setting that maintains a Vt of 6 mL/kg IBW, plateau pressure < 30 cmH_2_O, and ∆P < 15 cmH_2_O is recommended [20]. The findings of our present study were consistent with those of previous studies, in that increases in ∆P were strongly associated with increased mortality and adjusting ventilatory parameters to reduce ∆P may have a role in improving the outcomes of patients with early ARDS.

In patients with moderate to severe ARDS, a ∆P < 14 cmH_2_O may reflect improved lung compliance or an appropriate Vt/PEEP setting [4, 21]. Therefore, it is important to maintain a lower ∆P level, and continuous monitoring of ∆P, as opposed to monitoring only on Day 1, is recommended. In this study, we continuously monitored the serial changes in ∆P and found that 60-day survival significantly differed among the four patient groups. According to our study, failure to maintain the ∆P of patients with ARDS at a lower level for the first few days could lead to significantly higher mortality. Thus, the Day 1 ∆P level alone is insufficient to predict the outcomes of patients with ARDS.

This study has several limitations. First, this was a retrospective observational study performed in a single hospital center. Because of the retrospective nature of the study, some factors that might have affected survival, e.g., use of corticosteroids, appropriate antibiotic treatment, microbiology and superinfections, could not be counted. Second, the data regarding ventilator parameters, including ∆P, were not protocolized. Therefore, measurement bias cannot be ruled out. Third, we did not directly measure transpulmonary ΔP, which could better reflect lung parenchymal stress. Finally, the clinicians attempted different methods to reduce high ΔP, and the clinical relevance of each method was not evaluated or stratified. Further large-scale prospective studies are warranted to confirm the applicability of our findings in clinical practice.

## Conclusions

Driving pressure remains an important factor that predicts the survival of patients with ARDS. Continuous monitoring of ∆P, as opposed to monitoring only on Day 1, is recommended. Low ∆P should be maintained throughout early ARDS to improve patient survival.

## Data Availability

The datasets used and/or analyzed during the current study are available from the corresponding author on reasonable request.

## Abbreviations

ARDS: Acute respiratory distress syndrome
ICU: Intensive care unit
ECMO: Extra-corporeal membrane oxygenation
∆P: Driving pressure
Vt: Tidal volume
IBW: Ideal body weight
PEEP: Positive end-expiratory pressure
aHR: Adjusted hazard ratio

## References

[1] ARDS Definition Task Force (2012). Acute respiratory distress syndrome: the berlin definition. JAMA.

[2] Ashbaugh D, Boyd Bigelow D, Petty T, Levine B (1967). Acute respiratory distress in adults. Lancet.

[3] Ware LB, Matthay MA (2000). The acute respiratory distress syndrome. N Engl J Med.

[4] Bellani G, Laffey JG, Pham T, Fan E, Brochard L, Esteban A (2016). Epidemiology, patterns of care, and mortality for patients with acute respiratory distress syndrome in Intensive care units in 50 countries. JAMA.

[5] Fan E, Brodie D, Slutsky AS (2018). Acute respiratory distress syndrome: advances in diagnosis and treatment. JAMA.

[6] Petrucci N, Iacovelli W (2007). Lung protective ventilation strategy for the acute respiratory distress syndrome. Cochrane Database Syst Rev.

[7] Amato MBP, Barbas CSV, Medeiros DM, Magaldi RB, Schettino GP, Lorenzi-Filho G (1998). Effect of a protective-ventilation strategy on mortality in the acute respiratory distress syndrome. N Engl J Med.

[8] Amato MBP, Meade MO, Slutsky AS, Brochard L, Costa ELV, Schoenfeld DA (2015). Driving pressure and survival in the acute respiratory distress syndrome. N Engl J Med.

[9] Chiumello D, Carlesso E, Brioni M, Cressoni M (2016). Airway driving pressure and lung stress in ARDS patients. Crit Care.

[10] Baedorf Kassis E, Loring SH, Talmor D (2016). Mortality and pulmonary mechanics in relation to respiratory system and transpulmonary driving pressures in ARDS. Intensive Care Med.

[11] Chiu L-C, Hu H-C, Hung C-Y, Chang C-H, Tsai F-C, Yang C-T (2017). Dynamic driving pressure associated mortality in acute respiratory distress syndrome with extracorporeal membrane oxygenation. Ann Intensive Care.

[12] Bourenne J, Hraiech S, Roch A, Gainnier M, Papazian L, Forel J-M (2017). Sedation and neuromuscular blocking agents in acute respiratory distress syndrome. Ann Transl Med.

[13] Griffiths MJD, McAuley DF, Perkins GD, Barrett N, Blackwood B, Boyle A (2019). Guidelines on the management of acute respiratory distress syndrome. BMJ Open Respir Res.

[14] Blondonnet R, Joubert E, Godet T, Berthelin P, Pranal T, Roszyk L (2019). Driving pressure and acute respiratory distress syndrome in critically ill patients. Respirology.

[15] Guérin C, Papazian L, Reignier J, Ayzac L, Loundou A, Forel J-M (2016). Effect of driving pressure on mortality in ARDS patients during lung protective mechanical ventilation in two randomized controlled trials. Crit Care.

[16] Chen Z, Wei X, Liu G, Tai Q, Zheng D, Xie W (2019). Higher vs. lower DP for ventilated patients with acute respiratory distress syndrome: a systematic review and meta-analysis. Emerg Med Int.

[17] Bellani G, Grassi A, Sosio S, Gatti S, Kavanagh BP, Pesenti A (2019). Driving pressure is associated with outcome during assisted ventilation in acute respiratory distress syndrome. Anesthesiology.

[18] Bugedo G, Retamal J, Bruhn A (2017). Driving pressure: a marker of severity, a safety limit, or a goal for mechanical ventilation?. Crit Care.

[19] Pereira Romano ML, Maia IS, Laranjeira LN, Damiani LP, de MoraesPaisani D, de Carvalho Borges M (2020). Driving pressure–limited strategy for patients with acute respiratory distress syndrome. A pilot randomized clinical trial. Ann Am Thorac Soc.

[20] Sweeney RM, McAuley DF (2016). Acute respiratory distress syndrome. Lancet.

[21] Tsolaki VS, Zakynthinos GE, Mantzarlis KD, Deskata KV, Papadonta ME, Gerovasileiou ES (2021). Driving pressure in COVID-19 acute respiratory distress syndrome is associated with respiratory distress duration before intubation. Am J Respir Crit Care Med.

